# The Prevalence of Tooth Wear and Their Associated Etiologies Among Adult Subjects Visiting Umm Al-Qura University Dental Clinic in Makkah City, Saudi Arabia

**DOI:** 10.7759/cureus.59622

**Published:** 2024-05-04

**Authors:** Sahar M Elmarsafy, Wahdan M Elkwatehy, Rehab E Radi, Aseel K Alhindi, Raghad M Iskandar, Rahaf A Salem

**Affiliations:** 1 Restorative Dentistry, Faculty of Dental Medicine, Umm Al-Qura University, Makkah, SAU; 2 Conservative Dentistry, Faculty of Dental Medicine for Girls, Al-Azhar University, Cairo, EGY; 3 Dental Public Health and Preventive Dentistry, Faculty of Dentistry, Mansoura University, Mansoura, EGY; 4 Dentistry, King Faisal Hospital Makkah, Makkah, SAU; 5 Dentistry, Private Dental Clinic, Makkah, SAU

**Keywords:** etiologies, erosion, abrasion, attrition, tooth wear

## Abstract

In the past two decades, changing trends in socioeconomic status, dietary habits, and individual lifestyles of individuals have led to the emergence of tooth wear as an oral health problem. The present study aimed to investigate the prevalence and the associated etiologies of tooth wear in a convenience sample of adult patients visiting outpatient clinics of the Faculty of Dentistry at Umm Al-Qura University. This cross-sectional study was conducted on adult patients (18-40 years old) visiting outpatient clinics of the Faculty of Dentistry, Umm Al-Qura University. Two trained examiners visually assessed patients’ tooth wear using Smith and Knight’s Tooth Wear Index (TWI). Following the clinical examination, patients completed a self-administered questionnaire detailing risk factors such as the frequency of intake of acidic food and medicines, general health, chewing habits, dietary factors, and oral health-associated preventive behaviors. The resulting collected data were tabulated and statistically analyzed using Statistical Product and Service Solutions (SPSS, version 21; IBM SPSS Statistics for Windows, Armonk, NY). The total prevalence of tooth wear was 74%, and the recorded mean wear score (TWI) was 0.380 ± 0.386; anterior teeth exhibited greater wear than posterior teeth. Numerous associations were recorded between tooth wear and the tested variables in demographics, habits, diet, and medications, but most of them were not statistically significant. When toothbrushing habits were explored, the only factors to played a significant role in abrading the tooth structure were the type of brush bristles used (P-value = 0.026) and the frequency of brush renewal (P = 0.043). Patients who frequently ate citrus fruits and other acidic foods recorded high wear scores (0.509 ± 0.311 and 0.508 ± 0.402, respectively), although the difference was not statistically significant. When chewing occurred on both sides of the mouth, less tooth wear was recorded than if chewing was on the right or left side only (0.371 ± 0.260, 0.422 ± 0.273, and 0.520 ± 0.419, respectively). The study data support an association between tooth wear and patient occupation, use of hard-bristled and new toothbrushes, eating of citrus and other acidic food, and chewing on one side, as all of these factors increased the risk of tooth wear.

## Introduction

Tooth wear describes the non-carious loss of tooth tissue as a result of the interaction of three processes, which may occur in isolation or in combination: attrition, erosion, and abrasion. Attrition is the loss of tooth substance or a restoration caused by mastication or contact between occluding surfaces, while erosion is the progressive loss of hard dental tissues by chemical processes not involving bacterial action. Abrasion, by contrast, is the pathological loss of tooth substance caused by abnormal and repetitive mechanical wearing other than tooth-to-tooth contact [1].

The occurrence of tooth wear is known to be caused by numerous factors, resulting in different patterns of wear, which often occur concurrently, thereby rendering analysis and management highly complex [2]. A study conducted in 2023 revealed several significant variables that contribute to tooth wear, including parafunctional activities (69.7%), gastrointestinal disorders (60.5%), food (44.7%), foreign objects (19.9%), and missing teeth (9.2%) [3].

Acidic foods and drinks are known to play a major role in the progress of tooth wear. A considerable body of academic research indicates that low-pH foods and drinks cause erosion of enamel and dentine; however, clinical evidence is less convincing [4]. Most studies on children and adolescents support the finding that acidic foods and drinks cause erosive tooth wear, but comparatively few have assessed these risk factors in adults [5,6]. Another recognized risk factor is gastric acids, presenting as regurgitation or vomiting [3].

Tooth wear frequently causes discomfort and sensitivity, particularly when eating, drinking, or brushing teeth. If left untreated, it may lead to pain or the tooth being non-vital. Tooth wear is a complex and irreversible process influenced by multiple factors. Managing this problem is challenging, and early detection of wear is crucial. Neglecting to diagnose wear in its early stages can result in ongoing loss of tooth tissue and ultimately failure of the restoration process [7].

Tooth wear has attracted increased interest in dental research. Although caries rates are down in advanced economies, several authors describe a growing tendency toward tooth wear among young individuals. While there is ample information available on the prevalence of tooth wear in children and adolescents [4,5], there is a lack of comprehensive and organized data regarding adults, as well as little information regarding the natural progression of tooth wear despite anecdotal reports of its prevalence in adults based on clinical encounters. Tooth wear indices are the only valid and reliable method for assessing changes to teeth in wide populations [8]. The majority of indices use alterations in the anatomical structure of teeth to measure the extent of tooth wear. Various indices assess tooth wear on all surfaces of every tooth, while others focus on selected sites or specialized surfaces. The challenge lies in accurately diagnosing the real cause of tooth wear and employing an index that specifically excludes other potential causes. Identifying the cause of a lesion solely based on its appearance is clinically challenging, especially in the absence of an extensive dietary and dental history [9].

Accordingly, the aim of this study was to investigate the prevalence and associated etiologies of tooth wear in a convenience sample of adult patients visiting outpatient clinics of the Faculty of Dentistry at Umm Al-Qura University in Makkah City, Saudi Arabia.

## Materials and methods

Study design

This is a cross-sectional study conducted on adult patients (18-40 years old) visiting outpatient clinics of the Faculty of Dentistry, Umm Al-Qura University in Makkah City, Saudi Arabia. Ethical approval was obtained from the Institutional Biomedical Research Ethics Committee of Umm Al-Qura University (IRB approval no. HAPO-02-K-012-2021-11-828 & date 9/11/2021). The study employed a stratified sampling method, with the sample size estimated based on the expected prevalence. The size of the sample was calculated using the ClinCalc program (http://clincalc.com/stats/samplesize.aspx). For a 5% acceptable margin of error and an alpha level of 0.05, assuming that the prevalence of tooth wear in the 18-40-year-old group was 51% with an 85% confidence interval (CI), the sample size required was 182 subjects [10].

Sample selection

The study was open to individuals of either gender between the ages of 18 and 40 years who were willing to cooperate and sign the relevant informed consent letter. The following conditions were grounds for exclusion: presence of orthodontic bands/bracket, presence of crowns, presence of removable partial dentures, open bite malocclusion, tumor(s) of the soft or hard tissues of the oral cavity, active periodontal disease, developmental disorders, or retained primary teeth [11,12]. Each participant signed an informed consent letter before enrolling in the study.

Clinical examination

Two trained examiners conducted the clinical examination. Before the actual examination, they calibrated utilizing a set of samples within guidance. The inter-examiner and intra-examiner kappa values were calculated and found adequate. Both examiners achieved inter-examiner kappa scores exceeding 0.70 after training, although their intra-examiner kappa values were 0.80 and 0.81.

The clinical examination was done on the dental chair using routine oral examination instruments (i.e., a regular mouth mirror and an exploratory probe). All the surfaces of the teeth, except for the third molar, were assessed for lesions, including the buccal, cervical, occlusal/incisal, and lingual surfaces. Both examiners conducted wear assessments on the tooth surfaces after drying them with cotton rolls, using artificial light without applying amplification. Tooth wear assessment of all teeth was conducted using Smith and Knight’s Tooth Wear Index (TWI) [13] (Table 1). The TWI is an extensive method that assesses the wear on all four apparent surfaces (buccal, cervical, lingual, and occlusal/incisal) of all teeth. The third molar and restored or carious teeth were excluded from the analysis. Scores of 0-4 were assigned according to the severity of wear.

**Table 1:** Smith and Knight tooth wear index (TWI) B: buccal; L: lingual, O: occlusal; I: incisal; C: cervical. Table data extracted from [13].

Score	Surface	Criteria
0	B/L/O/I	No loss of enamel characteristics.
	C	No loss of contour.
1	B/L/O/I	Loss of enamel surface characteristics.
	C	Minimal loss of contour.
2	B/L/O	Loss of enamel exposing dentine for less than one-third of the surface.
	I	Loss of enamel just exposing dentine.
	C	Defect less than 1 mm deep.
3	B/L/O	Loss of enamel exposing dentine for more than one-third of the surface.
	I	Loss of enamel and substantial loss of dentine.
	C	Defect less than 1–2 mm deep.
4	B/L/O	Complete enamel loss—pulp exposure—secondary dentin exposure.
	I	Pulp exposure or exposure of secondary dentine.
	C	Defect more than 2 mm deep—pulp exposure—secondary dentine exposure.

Questionnaire

Following the clinical examination, the examiners administered a questionnaire to each participant. A specially customized questionnaire was designed for the study, and the items (questions) included within it were extracted from previous scientific sources [2,11,12]. The questions were asked in Arabic and covered the frequency of acidic food intake (fresh fruit, fruit juices, vegetable juices, carbonated drinks, yogurt, coffee, wine, pickled vegetables, vinegar); consumption of medications such as vitamin C, aspirin, amphetamines, and diazepam; general health concerns such as symptoms of reflux, vomiting, and eating disorders; digestive system diseases such as gastro-esophageal reflux disease, gastritis, and xerostomia; frequency of swimming in the summer; chewing habits; dietary factors; oral health-related preventive behaviors; and family’s socioeconomic class. The interviewer asked the participants to answer each question with the appropriate response.

Statistical analysis

Statistical Product and Service Solutions (SPSS, version 28.0; IBM SPSS Statistics for Windows, Armonk, NY) software was utilized to carry out the statistical analysis. Means and standard deviation (SD) were computed for the quantitative variables such as number of teeth and score of tooth wear index, whereas the frequency distribution of the qualitative variables was determined. Because the TWI data at the subject level were ordinary, mean tooth wear scores were calculated for the purpose of comparing between groups. A one-way ANOVA test was used to investigate the relationship between tooth wear scores and the investigated factors; comparing among more than two variables, while an independent sample t-test was applied to compare between two variables. The significance level was set at P = 0.05.

## Results

Demographics

A total of 182 participants were assessed; 116 were women, and 66 were men. Half of the participants (n = 91) were non-Saudi; however, more than 80% (n = 152) were born and raised in Saudi Arabia. All participants listed themselves as educated, but only 37% (n = 67) had jobs. All the questionnaire participants believed that they had a “good” health status. As for the socioeconomic section, only 6.7% (n = 12) answered “low,” with the majority lying in the middle status 80% (n = 146) (Table 2).

**Table 2:** The effect of demographic variables on tooth wear *The mean difference is significant at the P ≤ 0.05 level.

Variables	Groups	Prevalence, N (%)	Age (Mean ± SD)	P-value	Wear Score/TWI (Mean ± SD)	P-value
Nationality	Saudi	91 (50)	29.23 ± 8.72	0.514	0.586 ± 0.506	0.646
	Non-Saudi	91 (50)	30.93 ± 11.16		0.517 ± 0.627	
Place of birth	KSA	152 (83.5)	28.94 ± 8.62	0.046*	0.542 ± 0.593	0.770
	Outside KSA	30 (16.5)	35.80 ± 14.24		0.600 ± 0.428	
Parents’ nationality	Saudi	100 (55)	29.03 ± 8.54	0.370	0.394 ± 0.287	0.449
	Non-Saudi	82 (45)	31.37 ± 11.51		0.455 ± 0.334	
Educational level	Educated	182 (100)	30.08 ± 9.97	—	0.422 ± 0.308	—
	Noneducated	0 (0)	0.00 ± 0.00		0.000 ± 0.000	
Occupation status	Working	67 (36.8)	36.09 ± 11.45	0.000	0.535 ± 0.289	0.028*
	Not working	115 (63.2)	26.60 ± 7.07		0.356 ± 0.302	
Gender	Male	66 (36.3)	32.27 ± 10.68	0.198	0.563 ± 0.543	0.917
	Female	116 (63.7)	28.82 ± 9.44		0.546 ± 0.584	
Health status	Well	182 (100)	30.08 ± 9.97	—	0.422 ± 0.308	—
	Non-well	0 (0)	0.00 ± 0.00		0.000 ± 0.000	
Socio-economic status	High	24 (13.3)	32.50 ± 17.33	0.740	0.538 ± 0.332	0.443
	Moderate	146 (80)	29.77 ± 8.84		0.411 ± 0.313	
	Low	12 (6.7)	29.00 ± 3.37		0.317 ± 0.115	

Prevalence of tooth wear

**Figure 1:**
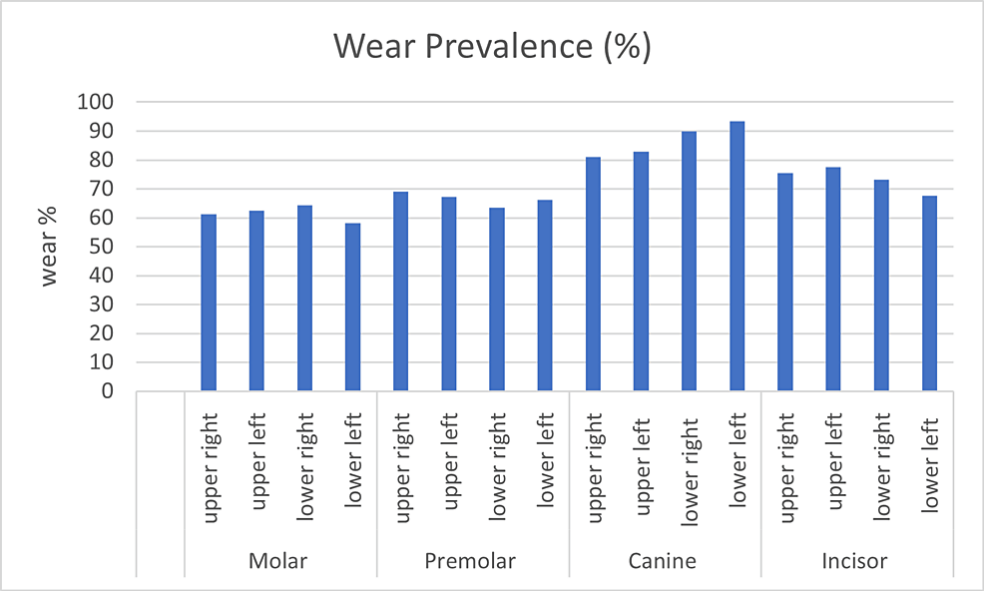
Bar chart showing tooth wear prevalence according to tooth position

The prevalence of tooth wear was found the highest in the lower left (n = 170, 93.3%) and right (n = 164, 90%) canines, followed by the upper left and right canines (n = 144, 83%; n = 141, 80%), respectively, whereas the teeth with least percentage of wear were found to be the lower left molars (n = 75, 58%). The total tooth wear prevalence was 74% (n = 130), and the recorded mean wear score (TWI) was 0.380 ± 0.386. No significant differences were recorded in wear scores between teeth (Figure 1, Table 3).

**Table 3:** Prevalence and mean score of tooth wear according to tooth position *The mean difference is significant at the p ≤ 0.05 level.

Tooth	Tooth position	Prevalence, N (%)	Wear Score/TWI (Mean ± SD)	One-way ANOVA
Molar	Upper right	90 (61.2)	0.270 ± 0.254	0.663
	Upper left	90 (62.5)	0.313 ± 0.307	
	Lower right	81 (64.3)	0.256 ± 0.224	
	Lower left	75 (58.1)	0.267 ± 0.252	
Premolar	Upper right	114 (69.1)	0.341 ± 0.338	0.964
	Upper left	111 (67.3)	0.313 ± 0.386	
	Lower right	105 (63.6)	0.327 ± 0.408	
	Lower left	111 (66.1)	0.313 ± 0.279	
Canine	Upper right	141 (81.0)	0.552 ± 0.565	0.338
	Upper left	144 (82.8)	0.491 ± 0.442	
	Lower right	164 (90.0)	0.521 ± 0.428	
	Lower left	170 (93.3)	0.496 ± 0.373	
Incisor	Upper right	129 (75.4)	0.434 ± 0.490	0.131
	Upper left	135 (77.6)	0.431 ± 0.403	
	Lower right	132 (73.3)	0.417 ± 0.356	
	Lower left	138 (76.7)	0.429 ± 0.345	
TOTAL		1930 (74.0)	0.380 ± 0.386	

Association between tooth wear and variables

When examining the association between tooth wear and different variables, such as demographics, habits, diet, and medications, numerous associations were recorded, but most of them were not statistically significant. The only demographic data point to show a significant relation to wear was the occupation of the individual p-value (0.028; Table 2). When toothbrushing habits were explored, the only factor to have played a significant role in abrading the tooth structure was the type of brush bristles used (p-value = 0.026) and the frequency of brush replacement (p = 0.043; Table 4). When considering dietary habits, participants who frequently consumed citrus fruits and foods containing acidic substances recorded TWI that were 0.509 ± 0.311 and 0.508 ± 0.402, although the difference is not statistically significant (Table 5). In this study sample, only a few participants had specific medical histories or were on the specific medications the researchers deemed of interest, so the effects of those were not accurately determined (Table 6). No statistically significant correlation was recorded between tooth wear and lifestyle and variables in dental-care habits; however, it was noted that, when chewing occurred on both sides, less tooth wear was recorded than if chewing was on the right or left side only; wear scores were 0.371 ± 0.260, 0.422 ± 0.273, and 0.520 ± 0.419, respectively (Table 7).

**Table 4:** Effect of tooth brushing (oral hygiene) variables on tooth wear * The mean difference is significant at the p ≤ 0.05 level.

Variables	Groups	Prevalence, N (%)	Wear Score/TWI (Mean ± SD)	P-value
Frequency of tooth brushing	Once daily	61 (33.5)	0.544 ± 0.343	0.079
	Twice daily	88 (48.3)	0.346 ± 0.165	
	More	33 (18.2)	0.3.99 ± 0.459	
Duration of tooth brushing	2 min or less	85 (46.7)	0.400 ± 0.259	0.613
	More than 2 min	97 (53.3)	0.441 ± 0.347	
Type of the brush	Soft	91 (50)	0.3.26 ± 0.162	0.026*
	Hard	52 (28.3)	0.573 ± 0.364	
	Moderate	39 (21.7)	0.422 ± 0.308	
Brushing technique	Vertical	21 (11.5)	0.288 ± 0.141	0.310
	Horizontal	24 (13.2)	0.345 ± 0.208	
	Mixed	137 (75.3)	0.456 ± 0.345	
Toothpaste	Fluoridated	118 (65)	0.406 ± 0.298	0.583
	Non-fluoridated	64 (35)	0.452 ± 0.329	
Tooth brushing method	Manual	146 (80)	0.424 ± 0.312	0.758
	Electric	9 (5)	0.297 ± 0.189	
	Both	27 (15)	0.450 ± 0.334	
Brush renewal	Less than 3 months	66 (36.3)	0.317 ± 0.170	0.043*
	More than 3 months	116 (63.7)	0.483 ± 0.352	

**Table 5:** Effect of dietary habit variables on tooth wear * The mean difference is significant at the p ≤ 0.05 level.

Variables	Groups	Prevalence, N (%)	Wear Score/TWI (Mean ± SD)	P-value
Eating citrus fruits	Never	27 (15)	0.270 ± 0.130	0.161
	Once or twice	100 (55)	0.416 ± 0.328	
	More than 2 times	55 (30)	0.509 ± 0.311	
Eating food containing acidic substances	Never	42 (23.3)	0.405 ± 0.300	0.231
	Once or twice	73 (40)	0.353 ± 0.180	
	More than 2 times	67 (36.7)	0.508 ± 0.402	
Acidic drinks	Never	51 (28.3)	0.451 ± 0.415	0.589
	Once or twice	49 (26.7)	0.467 ± 0.285	
	More than 2 times	82 (45)	0.377 ± 0.240	
Maintenance of acidic drinks in the oral cavity	Never	167 (91.7)	0.435 ± 0.317	0.247
	Sometimes	15 (8.3)	0.268 ± 0.090	
	Always	0 (0)	0.000 ± 0.000	
Eating citrus fruits frequency	Never	97 (53.3)	0.439 ± 0.322	0.731
	Rare	27 (15)	0.315 ± 0.163	
	Sometimes	55 (30)	0.440 ± 0.345	
	Always	3 (1.7)	0.516 ± 0.000	
Drinking acidic juice before sleep	Never	133 (73.3)	0.439 ± 0.318	0.890
	Rare	16 (8.7)	0.336 ± 0.171	
	Sometimes	30 (16.4)	0.392 ± 0.345	
	Always	3 (1.6)	0.375 ± 0.000	
Drinking acidic juice after sports	Never	155 (85)	0.433 ± 0.324	0.889
	Rare	15 (8.4)	0.390 ± 0.247	
	Sometimes	6 (3.3)	0.281 ± 0.133	
	Always	6 (3.3)	0.348 ± 0.073	
Tea intake	Less than 6/week	124 (68.3)	0.396 ± 0.303	0.340
	More than 6/week	58 (31.7)	0.478 ± 0.317	
Hard food intake	Never	30 (16.7)	0.467 ± 0.363	0.578
	Rare	46 (25)	0.384 ± 0.369	
	Sometimes	76 (41.6)	0.466 ± 0.299	
	Always	30 (16.7)	0.321 ± 0.131	

**Table 6:** Effect of medications and medical conditions (general health) variables on tooth wear * The mean difference is significant at the p ≤ 0.05 level. nr: no response

Variables	Groups	Prevalence, N (%)	Wear Score/TWI (Mean ± SD)	P-value
Dryness of the mouth	Never	124 (68.3)	0.375 ± 0.256	0.065
	Rare	18 (10)	0.315 ± 0.158	
	Sometimes	28 (15)	0.614 ± 0.434	
	Always	12 (6.7)	0.633 ± 0.471	
Vitamin C	Never	128 (70)	0.468 ± 0.340	0.354
	Rare	21 (11.7)	0.345 ± 0.181	
	Sometimes	21 (11.7)	0.279 ± 0.221	
	Always	12 (6.6)	0.322 ± 0.108	
Aspirin	Never	176 (96.6)	0.423 ± 0.313	0.926
	Rare	3 (1.7)	0.477 ± 0.000	
	Sometimes	(0)	0.000 ± 0.000	
	Always	3 (1.7)	0.313 ± 0.000	
Amphetamine & Diazepam	Never	103 (56.7)	0.398 ± 0.236	0.228
	Rare	37 (20)	0.514 ± 0.388	
	Sometimes	nr (nr)	0.00 ± 0.00	
	Always	nr (nr)	0.00 ± 0.00	
Esophageal regurgitation (reflex)	Never	143 (78.4)	0.401 ± 0.255	0.373
	Rare	18 (10)	0.518 ± 0.570	
	Sometimes	15 (8.3)	0.586 ± 0.411	
	Always	6 (3.3)	0.211 ± 0.773	
Vomiting	Never	161 (88.3)	0.438 ± 0.318	0.527
	Rare	18 (10)	0.310 ± 0.205	
	Sometimes	3 (1.7)	0.234 ± 0.00	
	Always	0 (0)	0.000 ± 0.00	
Stomach-ectomy	No	179 (98.3)	0.425 ± 0.309	0.544
	Yes	3 (1.7)	0.234 ± 0.00	

**Table 7:** Effect of lifestyle and dental habit variables on tooth wear * The mean difference is significant at the p ≤ 0.05 level.

Variables	Groups	Prevalence, N (%)	Wear Score/TWI (Mean ± SD)	P-value
Swimming	Rare	161 (88.3)	0.424 ± 0.315	0.483
	1–2/week	18 (10)	0.347 ± 0.232	
	More than 2/week	3 (1.7)	0.750 ± 0.00	
Teeth friction	Never	130 (71.3)	0.426 ± 0.290	0.751
	Rare	15 (8.4)	0.306 ± 0.239	
	Sometimes	34 (18.5)	0.470 ± 0.412	
	Always	3 (1.8)	0.00 ± 0.00	
Bruxism	Never	133 (73.3)	0.439 ± 0.306	0.786
	Rare	15 (8.3)	0.288 ± 0.216	
	Sometimes	15 (8.3)	0.427 ± 0.268	
	Always	19 (10)	0.406 ± 0.443	
Nail biting	No	149 (81.7)	0.441 ± 0.302	0.306
	Yes	33 (18.3)	0.335 ± 0.333	
Chewing side	Right	58 (31.7)	0.422 ± 0.273	0.347
	Left	43 (23.3)	0.520 ± 0.419	
	Both	81 (45)	0.371 ± 0.260	

## Discussion

The tooth wear prevalence rate of 74% recorded in this study sample of adult patients visiting the outpatient clinics of the Faculty of Dentistry, Umm Al-Qura University in Makkah City, Saudi Arabia, indicates that a significant majority exhibited some degree of tooth wear. Several factors may contribute to a high prevalence of tooth wear in a given population or clinical setting; these factors can include dietary habits (consumption of acidic and abrasive foods and beverages), oral hygiene practices, cultural habits, socioeconomic factors, the presence of underlying medical conditions, and the use of certain medications [5,14]. The prevalence of tooth wear exhibits considerable heterogeneity among the general public, and a comprehensive understanding of the global prevalence of tooth wear remains elusive. Researchers have conducted extensive investigations into the occurrence of distinct types of tooth wear on a global scale; these studies have reported varying prevalence rates, ranging from as low as 7% to as high as 84% [7,15].

Numerous research studies have revealed convincing associations between tooth wear and advancing age, underscoring that the intensity of tooth wear amplifies with increasing age [2,16]. Furthermore, the demographic trend toward an aging population has led to a heightened incidence of natural tooth retention, consequently contributing to an elevated prevalence of tooth wear among elderly individuals. A study done in the Eastern province of Saudi Arabia reported a prevalence of tooth wear of 83.5% in the adult population, which is consistent with the results of this study [17].

The observed diversity in reported tooth wear prevalence can be attributed to factors, including geographical variances, sample sizes, the utilization of different indices for measuring wear, the specific type of tooth wear under investigation, and age group.

The highest prevalence of tooth wear was identified in the lower canines, with slightly lower but still significant prevalence observed in the upper right and left canines. These findings are in agreement with the study conducted by Liu et al. [2], which detected a prevalence rate of 100% for tooth wear in both the mandibular and maxillary canines. Different groups of maxillary teeth had different rates, with molars at 85.51%, premolars at 89.77%, and incisors at 87.22%. The corresponding rates in the mandibular teeth for the three categories were 86.36%, 88.92%, and 91.19%, respectively. However, there was no significant variation in the maxilla or mandible between the categories [2]. Another study whose results are consistent with the results of this study was that of Schierz et al. [16] who found that tooth wear was, on average higher, for anterior teeth than for posterior teeth.

On the other hand, the results are inconsistent with those of Ali et al. [11], who reported that non-carious tooth wear mainly affects premolars and molars, whereas incisors are the least affected teeth. Occlusal and incisal were the most affected areas, followed by the cervical surface, and the least frequency of lesions was on the lingual surface [1]. The study of Ali et al. [11] is another study that found contradicting results; they found that the most affected tooth wear was recorded in mandibular molars (15.8%), followed by maxillary incisors (8.8%), then mandibular incisors (5.3%), and maxillary molars (2.6%). They took into account the limitation of the study as they only recorded tooth wear on molars and incisors, while the premolars and canines were not included in the evaluation and analysis [11].

The following are some of the possible causes of the noticeable increase in wear on the incisors and canines: incisors are smaller and have thinner enamel; when compared to the bigger posterior teeth, the incisors and canines may be subjected to higher pressures due to their active participation in chewing and jaw motions during parafunction and function; and the amount of wear that older persons suffer may be influenced by the fact that incisors and canines are the most often retained teeth within this age group [2]. Moreover, the canines, both upper and lower, typically have more prominent and pointed cusps compared to molars. As such, they may play a more active role in biting and tearing food, resulting in greater exposure to wear-inducing factors such as mechanical forces and dietary abrasives [18]. The lower canines, in particular, are positioned in the front of the mouth and may have increased exposure to abrasive substances and mechanical forces during activities such as chewing and grinding [19].

The prevalence of tooth wear can also be influenced by individual habits such as bruxism (teeth grinding) or clenching, and these habits may disproportionately affect certain teeth, leading to more wear in those areas [20]. Dietary factors, including the consumption of acidic or abrasive foods and beverages, can impact tooth wear, and canines, being involved in initial food breakdown and preparation for digestion, may be exposed to these factors to a greater extent [21,22].

By contrast, the teeth exhibiting the lowest percentage of wear were the lower molars, with only 58% showing signs of wear. This may be because the lower molars, being further back in the mouth, receive some protection from the effects of wear due to their position and the presence of adjacent teeth [23].

In this study, an association was observed between tooth wear and occupation status. The working participants recorded significantly more tooth wear, which is in accordance with a previous study that found a positive association between erosion and employment and reported that tooth wear is significantly higher in blue-collar workers [24]. There is no clear reason why occupation is related to tooth wear and erosion; we may speculate that it is due to the influence of a combination of occupational factors, lifestyle choices, and socioeconomic disparities [25].

This study also found tooth wear to be significantly higher in individuals who used harder toothbrush bristles. Evidence shows that hard bristles have a higher potential to cause enamel abrasion and can exacerbate existing tooth erosion [26,27]. Hard-bristle toothbrushes are generally discouraged, especially for individuals at risk of enamel erosion or those with sensitive teeth and gums [28].

Another remarkable finding in our study was the association of tooth wear with a higher intake of citrus fruits and tea. Citrus fruits, such as oranges and lemons, are acidic and can have erosive effects on tooth enamel when consumed frequently or in high quantities. The acid can soften enamel, making it more susceptible to wear. Numerous scientific studies have investigated the erosive effects of citrus fruits on tooth enamel. One study assessed the erosive potential of various fruit juices, including citrus juices, and found that citrus juices were among the most erosive beverages tested, causing significant enamel surface softening [4]. In situ studies have shown that frequent consumption of citrus fruits or their juices over an extended period can lead to surface texture changes and enamel loss, indicating the erosive potential of these fruits [6]. Tea, particularly acidic varieties such as black tea, can also contribute to tooth erosion due to the beverage’s acidity. Evidence shows that frequent and prolonged exposure to acidic beverages such as tea can soften and wear down tooth enamel over time [29].

Tooth wear can occur in individuals because of their health problems or the medications they take, which can cause the loss of salivary protection for the teeth. Thousands of drugs are acidic or have the ability to stop saliva production. It is common to find that alcohol, dehydration, systemic conditions, or medications can produce dry mouth (xerostomia) and sialadenosis as adverse effects. Some iron supplements, chewing vitamins C and aspirin, asthma inhalers, antihistamines, hypertension beta-blockers, antidepressants, sedatives such as amphetamine and diazepam, and chemotherapy drugs are among the most caustic medicines. In this study, we selected four high-risk medications (vitamin C, aspirin, amphetamine, diazepam) to investigate their association with tooth wear, and this selection was adopted from previous literature’s sources [7,12].

The scientific literature indicates that vitamin C is more corrosive than phosphoric acid and citric acid, dropping the pH of saliva below 5.5 causes the enamel to dissolve, and this effect can last for up to 25 minutes [15]. Aspirin, too, can potentially contribute to tooth erosion if it comes into direct contact with teeth; acetylsalicylic acid, the active ingredient, is a medication with a low pH. Some people chew or hold aspirin against their teeth to relieve pain, which can lead to localized erosion [30]. 

However, in this study, the effective relationship between aspirin use and tooth erosion was not proven, which is incompatible with other studies that recorded the highly significant effect of aspirin on tooth wear [5,12,15]. Zhang et al. [12] reported that, although all investigated medicines (vitamin C, aspirin, amphetamine, diazepam) were effective as a risk factor for tooth wear, only aspirin is significant. A total of 720 participants were enrolled in Wei et al.'s [15] investigation, which revealed associations between the percentages of tooth wear and dentin exposure and various factors, and they concluded that vitamin C and aspirin tablets were significantly associated with the development of erosion.

In cases where a high prevalence of tooth wear is observed, it is essential for dental professionals to provide appropriate education, preventive measures, and treatment options to help manage and mitigate further tooth wear. This might include recommendations for dietary modifications, the use of protective appliances such as mouthguards, and strategies to improve oral hygiene.

Limitations

The study’s sample population consists of adult patients visiting a single dental clinic, and this may not represent the broader population as it focuses solely on those seeking dental care at that particular facility. Patients who visit a dental clinic may have different oral health needs and behaviors compared to those who do not seek dental care regularly. This selection bias could affect the prevalence rates of tooth wear observed in the study. Additionally, the study appears to be cross-sectional, providing a snapshot of tooth wear prevalence at a specific point in time. It may not capture changes or trends in tooth wear over time, and causality cannot be determined from cross-sectional data. Moreover, the study’s findings may rely on self-reporting by patients or clinical examinations, which can introduce subjectivity and potential reporting bias. Patients may not accurately recall or report their dietary habits or behaviors related to tooth wear.

Age is a significant determinant of tooth wear in individuals of all age groups, including children, adolescents, adults, and the elderly. Therefore, it is necessary to perform comprehensive studies on this topic. Assessment of age factor is one of the shortcomings of this study as the target was only the adult age (18-40 years), and although the ages of all participants in the study were recorded, its association with tooth wear was not statistically analyzed. Additionally, the association between other factors such as gender, nationality, place of birth, and tooth wear was not fully explained within this study. The study may not fully capture all potential risk factors for tooth wear, which can limit the ability to identify specific causes or associations. There was a lack of assessment and interpretation between the different types of tooth wear (attrition, abrasion, erosion, and abfraction); additionally, some biological variables were overlooked, including saliva, the composition and structure of teeth, and the nature of occlusion. All of these variables are recommended to be included in future studies.

## Conclusions

The prevalence of tooth wear among 18-40-year-old adult patients of the dental clinic at Umm Al-Qura University in Makkah City was 74%, in which the anterior teeth exhibit greater wear than posterior teeth. Tooth wear is a common multifactorial disease, and the study data support the associations between tooth wear and occupational status, the use of hard and new toothbrushes, eating acidic foods such as citrus, and chewing on one side, all of which increased the risk of tooth wear. Prevention and awareness among the population about tooth wear need to be enhanced, and emphasis on the importance of good dietary intake and daily habits is essential.

## Appendices

**Table 8:** Study questionnaire items The questionnaires are in both Arabic and English languages.

The Prevalence of Tooth Wear and Their Associated Etiologies Among Adult Subjects Visiting Umm Al-Qura University Dental Clinic in Makkah City, Saudi Arabia - Study Questionnaire Items							
معلومات عامة General & demographic information:							
اسم المريض Patient name							
جهة اتصال شخصية Personal contact							
ملف المريض # Patient file #							
الجنسية Nationality							
هل ولدت في السعودية Are you born in Saudi Araba	نعم Yes			لا اين If no, in which country			
من أي بلد الوالدين Which country are your parents from							
التعليم Education	غير متعلم Uneducated			متعلم Educated			
العمل Work	يوجد عمل Employed			لا يوجد عمل Unemployed			
العمر Age							
الجنس Sex	ذكر Male			انثى Female			
الحالة الصحية العامة General health status	جيدة Good			معتلة Sick			
الحالة الاجتماعية والاقتصادية Socio-economic status	منخفضة Low	متوسطة Moderate				عالية High	
عوامل نظافة الفم oral hygiene factors:							
كم مرة يتم تنظيف الاسنان Frequency of brushing	مرتين او أكثر يومياً Twice or more daily			مرة واحدة او اقل يومياً Once or less daily			
مدة تنظيف الأسنان بالفرشاة Duration of brushing	أكثر من 2 دقيقة more than 2 min			أقل من أو يساوى 2 دقيقة less than 2 min			
نوع فرشاة الأسنان Toothbrush bristles type	شعيرات ناعمة Soft		متوسطة الخشونة Medium				خشنة Hard
طريقة تفريش الأسنان Tooth brushing method	تفريش أفقي Horizontal brushing		تفريش رأسي Vertical brushing				تفريش مختلط Mixed brushing
هل يحتوي معجون الاسنان على الفلورايد Fluoride toothpaste	نعم Yes			لا No			
طريقة تنظيف الاسنان Tooth brushing type	فرشاة يدوية Manual toothbrush		فرشاة كهربائية Electric toothbrush				كلاهما Both
مدة تغيير فرشاة الأسنان Frequency of changing toothbrush	أكثر من 3 شهور More than 3 months			اقل من 3 شهور Less than 3 months			
العوامل الغذائية Dietary Factors:							
تناول الفاكهة الحمضية (الليمون، التفاح، البرتقال، العنب، جرب فروت، ....الخ) Eat citrus fruits (Lemon, apple, orange, grape, grapefruit, ....etc)	عدة مرات يوميا - مرة واحدة يومياً - أبداً several times a day - once a day - never						
تناول طعام يحتوي على حمضيات متنوعة (خل، مخللات، طعام بالكاري، الأعشاب والتوابل، صلصة السلطة، صلصة التغميس، الرنجة الحامضة، الزبادي، حلويات حامضة ...الخ) Eat a variety of citrus fruits (Vinegar, pickles, curry food, herbs and spices, salad dressing, dipping sauce, sour herring, yoghurt, sour dessert...etc.)	عدة مرات يومياً - مرة واحدة يومياً - أبداً several times a day - once a day - never						
تناول المشروبات الحمضية (كولا، مشروبات الطاقة، عصير الليمون، عصير البرتقال، عصير الفاكهة، مياه معلبة بنكهات حامضية ....الخ) Drink acidic drinks (Cola, energy drinks, lemonade, orange juice, fruit juice, citrus-flavored water....etc)	عدة مرات يومياً - مرة واحدة يومياً - أبداً several times a day - once a day - never						
طريقة شرب المشروبات الحمضية (الاحتفاظ بالمشروب لفترة داخل الفم قبل البلع أو مضمضة المشروب داخل الفم قبل البلع) How to drink acidic drinks (Holding the drink for a while in the mouth before swallowing or rinsing the drink inside the mouth before swallowing)	أبداً - أحياناً - غالباً never - sometimes - often						
شرب المشروبات الحمضية عن طريق المصاصة Drink acidic drinks through a straw	أبداً- نادراً- أحياناً - غالباً never - rarely - sometimes - often						
شرب المشروبات الحمضية قبل النوم Drink acidic drinks before bed	أبداً - نادراً - أحياناً - غالباً never - rarely - sometimes- often						
شرب المشروبات الحمضية مباشرة بعد الرياضة Drink acidic drinks immediately after exercise	أبداً - نادراً - أحياناً - غالباً never - rarely - sometimes - often						
عدد مرات استهلاك الشاي The number of times the tea is consumed	أقل من 6 مرة أسبوعياً - أكثر من 6 مرة أسبوعياً less than 6 times a week - more than 6 times a week						
تناول الطعام الصلب Eat solid food	أبداً - نادراً - أحياناً - غالباً never - rarely- sometimes - often						
عوامل الصحة العامة General Health:							
جفاف الفم Dry mouth	أبداً - نادراً- أحياناً - غالباً never - rarely - sometimes - often						
تناول مكملات فيتامين C Take Vitamin C Supplements	أبداً- نادراً - أحياناً - غالباً never - rarely - sometimes - often						
تناول الأسبرين Taking Aspirin	أبداً- نادراً -أحياناً- غالباً never - rarely - sometimes - often						
أخذ أدوية الأمفيتامين أو الديازيبام Taking Amphetamine & Diazepam	أبداً- نادراً - أحياناً - غالباً never - rarely - sometimes - often						
ارتجاع العصارة المعوية Reflux	أبداً - نادراً - أحياناً - غالباً never - rarely - sometimes - often						
التقيؤ Vomiting	أبداً - نادراً - أحياناً - غالباً never- rarely - sometimes - often						
اضطرابات الأكل Eating disorder	أبداً - نادراً - أحياناً - غالباً never - rarely- sometimes - often						
داء الارتداد المعدي المريئي Gastro esophageal reflux disease	أبداً - نادراً - أحياناً - غالباً never - rarely - sometimes - often						
استئصال جزء من المعدة Gastricism	نعم Yes				لا No		
عوامل نمط الحياة: Life Style factors							
تواتر (تكرار) السباحة في الصيف Frequency of swimming in summer	أبداً - نادراً - 1-2 مرة أسبوعياً - أكثر من 2 مرات أسبوعياُ never - rarely - 1-2 times/week - more than 2 times/week						
احتكاك الاسنان العلوية والسفلية تلقائياً Clenching teeth automatically	أبداً- نادراً - أحياناً - غالباً never - rarely- sometimes - often						
احتكاك الاسنان اثناء النوم Sleep bruxism	أبداً - نادراً - أحياناً - غالباً never - rarely - sometimes - often						
عادة عض الأظافر أو أشياء أخري صلبة Usually biting nails or other hard objects	نعم Yes			لا No			
جهة المضغ المعتادة Chewing habits	اليمين- اليسار- الجهتين right - left - both sides						
